# The Brazilian Cardiac Surgery, Although it has High International Prestige, Never Performed a Great "Trial"

**DOI:** 10.21470/1678-9741-2017-0509

**Published:** 2017

**Authors:** Domingo M. Braile, Paulo Roberto B. Évora

**Affiliations:** 1 Editor-in-Chief - BJCVS; 2 Editor-in-Chief Interim - BJCVS

## BJCVS in search of excellence

Doctor Herbert L. Fred^[1]^, an associate editor of the Texas Heart Institute Journal, pointed out five crucial components that make a successful medical journal: 1) financial security; 2) an ample, competent, and experienced editorial staff; 3) reliable manuscript reviewers; 4) equality of submissions, and; 5) responsive readers. In addition, one more fundamental component could be added, which would represent cardiac surgery, the ideas and contributions of the country. We are trying to follow these components to improve the excellence of our Brazilian Journal of Cardiovascular Surgery (BJCVS), encouraging the realization of substantial trials.

## A Great "Trial"

**Fig. 1:**
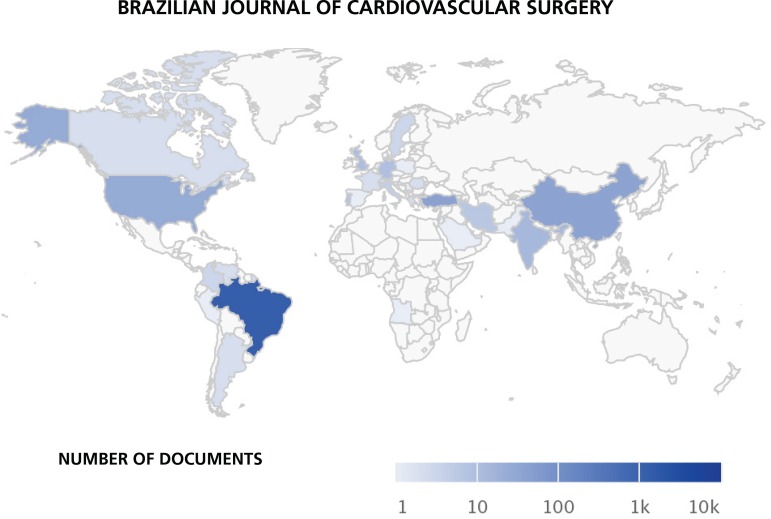
Distribution of the authors' affiliation countries.

The latest Sociedade Brasileira de Cirurgia Cardiovascular/Brazilian Society of Cardiovascular Surgery (SBCCV) newsletter (April 2017) highlights a consensus published by the American Heart Association (AHA) for the use of appropriate criteria for myocardial revascularization in stable angina^[2]^. This consensus is an update of the 2012 document, now divided into two publications, one referring to acute and present coronary syndrome. Unlike a standard guideline, this Consensus brings more than 60 real clinical settings, scored by a panel of 32 experts among clinicians, interventionists, and surgeons. The clinical, anatomical and functional characteristics were contemplated, and innovatively, the treatment with one or more antianginal drugs weighed in the intervention decision. This approach may be useful for an unambiguous standardization to correct regional discrepancies when, for example, EuroSCORE and STS are used. The Brazilian cardiac surgery, although it has high international prestige (Figure 1), never performed a great "trial." For this reason, the coronary artery bypass grafting without cardiopulmonary bypass would be chosen, since its introduction in the surgical practice was carried out by Dr. Enio Buffolo (in Brazil) and Dr. Federico Benetti (in Argentina).

(Source: SciELO)

## Articles in this Issue

This issue of BJCVS presents a blind peer-reviewed selection of 15 articles that will surely please your readers:

- Three papers related to congenital heart disease present and discuss outcomes of the superior cavopulmonary connection operation, evaluation of nosocomial infections in pediatric patients with extracorporeal membrane oxygenation support and neuroprotective anesthesia regimen and intensive management for pediatric cardiac surgery.
- Four presentations on risk factors: 1) hypothyroidism as risk factor for atrial fibrillation after coronary artery bypass graft; 2) validation of 4 prediction scores for cardiac surgeryassociated acute kidney injury in Chinese patients; 3) Brazilian pre-validation study of the disruptions in surgery index (DISI); and 4) B-type natriuretic peptide as a predictor of short-term mortality in on-pump CABG.
- Three articles on cardiac electrical stimulation: 1) an early experience on subcutaneous implantable cardioverter defibrillator; 2) analysis of dyssynchrony and ventricular function in the right univentricular stimulation; and 3) relationship between atrial fibrillation recurrence and brain natriuretic peptide (BNP) after successful electrical cardioversion.
- Two multimedia presentations: 1) spontaneous left anterior descending coronary artery dissection; and 2) embolization by bullet dislodged from the heart.
- Two case reports: 1) left atrial dissection, a rare cause of left ventricular assist device (LVAD) obstruction; and 2) stent graft relining in a patient with an acute aortic aneurysm and a wholly migrated endograft.
- One experimental study: comparison of arterial repair through suture, suture with fibrin or cyanoacrylate adhesive in *ex vivo* porcine aortic segment.
- One paper on outcome considering the effect of treatment strategy of chronic ischemic mitral regurgitation on long-term outcomes in coronary artery bypass grafting.
- One review on the left atrial appendage emphasizing issues that often are beyond of the cardiac surgeon's expertise.

**Domingo M. Braile** ^1^Editor-in-Chief - BJCVS **Paulo Roberto B. Évora** ^2^Editor-in-Chief Interim - BJCVS
